# A Korean single-center, real-world, retrospective study of first-line weekly paclitaxel in patients with metastatic angiosarcoma

**DOI:** 10.1186/s13569-016-0048-0

**Published:** 2016-05-05

**Authors:** Seonggyu Byeon, Haa-Na Song, Hee Kyung Kim, Jun Soo Ham, Su Jin Lee, Jeeyun Lee, Se Hoon Park

**Affiliations:** Division of Hematology-Oncology, Department of Medicine, Samsung Medical Center, Sungkyunkwan University School of Medicine, 81 Irwon-ro, Gangnam-gu, Seoul, 135-710 Korea; Department of Internal Medicine, Gyeongsang National University Hospital, Jinju, Korea

**Keywords:** Angiosarcoma, Chemotherapy, Paclitaxel, Retrospective

## Abstract

**Background:**

Angiosarcoma is a rare subgroup of soft tissue sarcomas associated with poor prognosis, but paclitaxel has been shown to be active in pretreated metastatic disease. We investigated the efficacy and safety of weekly paclitaxel as first-line chemotherapy in adult patients with metastatic angiosarcoma.

**Methods:**

A retrospective study using the Samsung Medical Center (Seoul, Korea) cancer chemotherapy registry was performed on 21 consecutive patients with angiosarcoma who were treated with weekly paclitaxel as first-line therapy for metastatic disease between Oct. 2008 and Dec. 2014. We excluded patients who were enrolled in clinical trials to ensure the results would reflect the real-world outcomes obtained in a daily clinical setting. Endpoints included efficacy in terms of response rate, progression-free survival (PFS), overall survival (OS) and safety.

**Results:**

Among 21 patients, 15 (71 %) were male and the median age was 53 years (range, 24–76). Primary sites of angiosarcoma were the visceral organs (33 %), scalp (29 %) and heart (23 %). The median number of metastatic sites was two (range, 1–5) with the lungs being the most frequently involved site. Weekly paclitaxel was generally well tolerated: the major hematologic toxicity was grade 1/2 anemia (24 %). Among non-hematologic toxicities, grade 1/2 peripheral neuropathy was most commonly observed (67 %). Objective response was observed in 11 (52 %) patients (4 complete and 7 partial responses). With a median follow-up of 21 months, the estimated median PFS and OS were 5.7 months (95 % CI 5.1–6.3) and 18.6 months (95 % CI 9.9–27.3), respectively.

**Conclusions:**

In this retrospective study, first-line chemotherapy with weekly paclitaxel demonstrated clinically relevant efficacy and tolerability in unselected Korean patients with metastatic angiosarcoma. It is encouraging that response rate and PFS for Korean patients were similar to those reported in Western reports.

## Background

Angiosarcoma is a rare (2 %) subgroup of soft tissue sarcoma (STS) derived from endothelial cell lineage associated with aggressive behavior and poor prognosis [1, 2]. When localized, complete surgical resection of tumors and adjuvant radiotherapy is the treatment of choice. However, surgery is often challenging, as some cases of angiosarcoma occur in the face or in other vital organs such as the heart and great vessels. According to a previous report [3], one-third of patients with localized angiosarcoma did not receive surgery because of its location and/or rapid progression. In general, the prognosis of angiosarcoma remains poor, even treatment with aggressive surgical resection is associated with a 5 year survival rate of 60 %, with median survival of 7 months [3]. Similar to other STS subtypes [4], conventional cytotoxic chemotherapy has shown limited success in angiosarcoma [5]. More than a few decades’ worth of clinical trials have indicated that cytotoxic chemotherapy with anthracyclines and/or ifosfamide is the standard for first-line treatment [6]. Unfortunately, with the exception of pazopanib in the second-line setting [7], clinical trials involving novel agents have had little success in the treatment of angiosarcoma. Nevertheless, it is of note that some retrospective studies have demonstrated that paclitaxel has favorable efficacy in angiosarcoma with improved survival [6, 8–10]. In a retrospective study written by Penel et al. [6], weekly paclitaxel given as first-line regimen was an independent parameter for improved survival. Cutaneous angiosarcoma responded more favorably to weekly paclitaxel than to doxorubicin [10].

Current guidelines recommend the use of paclitaxel, docetaxel, or all available systemic therapy options for STS as a first-line treatment for angiosarcoma [11]. Based on the results presented in the ANGIOTAX phase II study [12], as well as other retrospective studies described above, our institutionalstandard for first-line treatment of metastatic angiosarcoma has been weekly paclitaxel since 2008.

The choice of a first-line regimen for an individual patient may be important, since not all patients are eligible for salvage treatment, which obviously provides a rationale to first administer the most effective treatment. Factors to be considered include the experiences of the treating oncologists, potential toxicity, especially for those with symptoms or with decreased performance status, as well as the activity of chemotherapeutic agents. In prospective clinical trials including the ANGIOTAX [12], patients were selected on the basis of a fairly preserved performance status and normal organ function. Angiosarcoma is a highly aggressive disease that often exhibits rapid progression and clinical decline; therefore, the clinical trial population might not be indicative of all patients seen in daily oncology practice. In an effort to generate real-world data in Korean metastatic angiosarcoma patients, we conducted a retrospective review of a prospectively collected cancer chemotherapy registry. Although this study is limited by the retrospective nature of the analysis, the present evaluation was also done with the intent to develop improved therapeutic strategies for angiosarcoma patients with metastatic disease and support further prospective studies to better define the full therapeutic potential of paclitaxel.

## Patients and methods

Institutional Review Boards approval was obtained from Samsung Medical Center (SMC; Seoul, Korea). Written informed consent was given by all patients prior to starting chemotherapy, according to institutional standards. We retrospectively collected and reviewed the data of 21 adult patients with histologically-proven, metastatic angiosarcoma who were consecutively treated with weekly paclitaxel as first-line chemotherapy between Oct. 2008 and Dec. 2014. We excluded patients who were enrolled in clinical trials to ensure the study population reflected our daily clinical practice, and the choice of weekly paclitaxel was solely at the discretion of the treating oncologists. Other exclusion criteria for patient selection were as follows: (1) prior chemotherapy or targeted therapy for advanced or metastatic disease, (2) mixed histology with other STS subtypes, (3) another malignancy within 5 years, and (4) patients with inappropriate laboratory findings or severe comorbid illness for standard 80 mg/m^2^ weekly doses of paclitaxel. If adjuvant chemotherapy had been completed more than 6 months before the start of weekly paclitaxel, the patient could be included onto the study.

In all patients, chemotherapy was administered on an outpatient basis. Each chemotherapy cycle consisted of intravenous infusion of paclitaxel 80 mg/m^2^ over 1 h on days 1, 8 and 15. Premedications included dexamethasone 20 mg, chlorpheniramine 4 mg and ranitidine 50 mg intravenously. Other supportive care including the administration of anti-emetics, blood products, and the use of analgesics was given if judged appropriately by the treating physicians. Before initiating the first dose of chemotherapy, patients had a complete history taken, and underwent complete blood counts and serum chemistries, chest x-rays, and computed tomography scans of all involved sites. Patients were seen every 4 weeks as chemotherapy was repeated every 4 weeks. Therapy was continued until objective disease progression per Response Criteria in Solid Tumors (RECIST) [13], unacceptable toxicity or deterioration of clinical status, or patient refusal. Dose adjustments at the start of a new cycle were based on the worst toxicity observed during the previous cycle. For hematologic toxicity, subsequent cycles were delayed for up to 2 weeks if grade >2 toxicities appeared. The doses of paclitaxel were reduced if grade 4 myelosuppression or febrile neutropenia was present, and if myelosuppression or febrile neutropenia persisted an additional reduction of doses was required. If there was a third such episode, chemotherapy was discontinued. In case of Grade ≥2 non-hematologic toxicity, treatment was delayed until recovery but not for more than 2 weeks. Doses were reduced in the case of severe non-hematologic toxicity that was not controllable with usual measures, and treatment was discontinued if the patient experienced a significant hypersensitivity reaction or unacceptable toxicity (e.g., Grade 4 stomatitis or diarrhea, Grade ≥3 peripheral neuropathy, severe and persistent skin/nail changes). Chemotherapy administration could be omitted if one of the following toxicities was noted on day 8 or day 15: Grade ≥2 hematologic or non-hematologic toxicities, fever ≥38 °C, diarrhea of any grade, or a decrease in performance status.

Baseline characteristics and outcome data were collected using a uniform case report form. Clinical and laboratory parameters collected at the time of starting chemotherapy included age, gender, Eastern Cooperative Oncology Group (ECOG) performance status, histological grade according to Fédération Nationale des Centres de Lutte Contre le Cancer (FNCLCC), primary site, previous surgery and/or radiotherapy, blood counts and chemistries, time between diagnosis and paclitaxel therapy, and sites of metastases. Responses were evaluated every two cycles by chest and abdominopelvic computed tomography or by the same tests that were used to stage initial tumors. Adverse events were collected and graded according to the National Cancer Institute criteria. To determine the causes of death, as well as therapy discontinuation, a structured medical record review was performed.

The primary end point was the response rate (RR). Secondary end points included progression-free survival (PFS), overall survival (OS), and toxicity profile. PFS and OS were calculated using the Kaplan–Meier method. To examine the impact of baseline parameters collected on PFS and OS, Cox proportional hazard model was used. Laboratory parameters and age were initially recorded as continuous variables, and evaluated as both continuous and categorical variables. The potential presence of interaction effects between baseline parameters was tested by defining product terms for the respective factors in a regression model. All P values were two-sided, with P < 0.05 indicating statistical significance. All analyses were performed using the R for Windows v2.11.1 software (R Core Team, Vienna, Austria; http://www.Rproject.org).

## Results

The SMC cancer chemotherapy registry identified 21 eligible patients who were treated with first-line weekly paclitaxel for metastatic angiosarcoma. Patient characteristics are given in Table 1. As shown, men constituted 71 % of the patients and the median age was 53 years (range 24–76). Four (19 %) patients had been treated with adjuvant chemotherapy, involving doxorubicin/ifosfamide (n = 2) and etoposide/ifosfamide/cisplatin (n = 2). Primary sites of angiosarcoma were visceral organs (33 %), scalp (29 %) and heart (23 %). Approximately 67 % of the patients had two or more metastatic disease sites, mostly involving the lungs, lymph nodes and bone. At the time of data collection, with a median follow-up duration of 21 months, 20 patients showed disease progression and 14 patients died.

**Table 1:** Baseline characteristics (n = 21)

	No. of patients	Percentage
Age, years		
Median (range)	53 (24–76)	
Gender		
Male	15	71
Female	6	29
ECOG performance status		
0	4	19
1	16	76
2	1	5
Primary site		
Scalp	6	29
Non-scalp		
Visceral organs (e.g., liver)	7	33
Heart	5	24
Skin	2	10
Breast	1	5
FNCLCC grade		
1	1	5
2	7	33
3	9	43
Unknown	4	19
Initial stage at diagnosis		
III	11	52
IV	10	48
Laboratory (mean, SD)		
Hemoglobin, g/dL	12.6 (2.0)	
Corrected calcium, mg/dL	9.3 (0.7)	
Lactate dehydrogenase, U/L	372 (243)	
Number of metastatic site(s)		
Median (range)	2 (1–4)	
Sites of metastases		
Lung	12	57
Lymph nodes	9	43
Bone	5	24
Spleen	3	14
Liver	1	5

*ECOG* Eastern Cooperative Oncology Group, *FNCLCC* Fédération Nationale des Centres de Lutte Contre le Cancer

All 21 patients were evaluable for efficacy and safety. A total of 102 4-week chemotherapy cycles were administered (median 5, range 1–9). All but one patient received at least two cycles of weekly paclitaxel chemotherapy. The most common reason for chemotherapy discontinuation was disease progression. Overall, first-line weekly paclitaxel was generally well tolerated, with anemia and peripheral neuropathy being the most commonly observed toxicities (Table 2). One patient died of causes that could have been related to chemotherapy. A 38 years-old woman with angiosarcoma arising from pericardium died due to pulmonary thromboembolism occurred in the midst of second cycle, with no clinical evidence of progression.

**Table 2:** Maximum grade toxicities per patient

	All grades, N (%)	Grades 3 or 4, N (%)
Anemia	5 (24 %)	2 (10 %)
Neutropenia	3 (14 %)	2 (10 %)
Thrombocytopenia	1 (5 %)	0
Infection	1 (5 %)	1 (5 %)
Nausea and vomiting	13 (62 %)	1 (5 %)
Anorexia	10 (48 %)	
Stomatitis	6 (29 %)	0
Diarrhea	4 (19 %)	0
Fatigue	10 (48 %)	0
Peripheral neuropathy	14 (67 %)	0
Allergic reaction	2 (10 %)	0

Objective responses to weekly paclitaxel were noted in 11 patients (RR 52 %, 95 % CI 32–74 %), including four complete responses. Stable disease was observed in 6 patients (29 %), leading to a disease control rate of 81 %. In order to explore predictive factors for clinical response to chemotherapy, we performed a logistic regression analysis with known clinical and laboratory parameters. However, RR was not significantly influenced by age, gender, performance status, FNCLCC grade, number and site of metastases, or baseline laboratory parameters. We also tested whether the development of clinical responses was modified by interaction between the effects of parameters; the first-level interaction term between these variables was entered into separate multivariate model but we found no interaction between them.

**Fig. 1:**
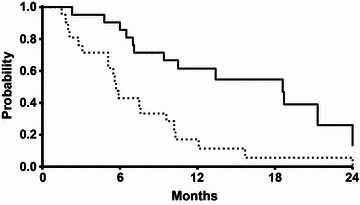
Kaplan–Meier curves for progression-free survival (*dotted line*) and overall survival (*solid line*)

Of the 21 patients analyzed in the study, the median PFS and OS were 5.7 months (95 % CI 5.1–6.3 months) and 18.6 months (95 % CI 9.9–27.3 months), respectively. The Kaplan–Meier estimates of PFS and OS are illustrated in Fig. 1. Again, in the Cox regression model, the estimated PFS or OS was not significantly influenced by any of the baseline parameters. After first-line failure, 38 % of the patients received second-line therapy, mostly with pazopanib (n = 7) and doxorubicin (n = 1). For exploratory purposes, we compared OS between patients who received salvage therapy after first-line paclitaxel failure and who did not. Although statistically insignificant, those who received second-line therapy lived longer (21.2 versus 13.3 months, P = 0.105).

## Discussion

In this retrospective study on a limited number of patients, we found that first-line therapy with weekly paclitaxel in Korean patients with metastatic angiosarcoma can be both well tolerated and active, regardless of performance status or other baseline characteristics. First-line weekly paclitaxel achieved objective response and stable disease in 52 and 29 % of patients, respectively. The estimated median PFS and OS were 5.7 months (95 % CI 5.1–4.3) and 18.6 months (95 % CI 9.9–27.3), respectively. Although this study is retrospective in nature, the results provide evidence that Korean patients with metastatic angiosarcoma may derive benefit from weekly paclitaxel. The results are compared well with the outcomes obtained from previous retrospective studies [6, 8–10], as well as the prospective phase II ANGIOTAX study [12].

While the mode of action of paclitaxel in patients with metastatic angiosarcoma remains unclear [12], and doxorubicin-based chemotherapy remains the treatment of choice in metastatic STS [14], weekly paclitaxel seems to be an active and safe chemotherapy regimen in patients with chemotherapy-naïve, metastatic angiosarcoma. Unfortunately, ANGIOTAX-PLUS [15], a randomized phase II study testing paclitaxel with or without bevacizumab, reported that the 6-month PFS rate was 56.7 % with weekly paclitaxel and 57.6 % with weekly paclitaxel plus bevacizumab. Although there are newer agents that can be used to treat metastatic STS, it is our conclusion that weekly paclitaxel should be offered in a first-line setting for patients with metastatic angiosarcoma. Paclitaxel can be administered to patients whose tumors have progressed after other agents such as doxorubicin. We believe this approach is not optimal because the patient’s performance status is likely to decline with each successive systemic treatment, and thus the opportunity to use paclitaxel can be lost.

Therefore, the identification of prognostic or predictive factors allowing the selection of patients who are likely to benefit from weekly paclitaxel is an important challenge. Our study showed that, among 21 patients with metastatic angiosarcoma, 4 (19 %) patients experienced progressive disease as their best response to weekly paclitaxel, which is comparable to findings in published reports. Although this study is retrospective in nature, it is clear that weekly paclitaxel may not be beneficial for all angiosarcoma patients with metastatic disease. In a large retrospective analysis [14], high tumor grade was an adverse prognostic factor for PFS and OS. We found that response to weekly paclitaxel was not related to the FNCLCC tumor grade or the performance status of patients. Besides clinical and laboratory parameters, appropriate patient selection based on molecular characterization of tumor is one of the most extensively studied area in clinical research. While it is still at an early stage and it would take years before we see clinical applications, extensive work is ongoing to identify possible molecular markers, particularly in the angiogenesis pathway. In patients with pretreated STS, pazopanib should be considered a treatment of choice based on a prospective phase III trial [7].

In the present study, 38 % of the patients received second-line therapy after the first-line paclitaxel failure. Considering the aggressive nature of the disease, it seems clear that only a small percentage of patients continue to have good performance status after first-line therapy and are still medically fit to be offered salvage therapy. Nevertheless, we cannot exclude the possibility that the favorable outcomes seen in our patient population is related to the effect of further therapy. The strength of this study includes its single-center nature with patients who were treated with weekly paclitaxel as first-line chemotherapy to avoid selection bias. It should be noted that our patient population was a consecutive series of patients taken from an academic, tertiary cancer center to reflect the real-world experience with first-line weekly paclitaxel. That is, the study reflects real-world outcomes that may not necessarily be seen in randomized controlled trials with selected patients. The limitations of this study include its retrospective nature, which may predispose the study to selection bias and issues with missing data. Selection bias can be minimized by obtaining consecutive series of patients, as seen in the current results. The lack of central radiology review, variable modalities of imaging, and intervals between scans are potential weaknesses; however, we believe this better reflects the real-world experience of oncologists using first-line chemotherapy. Finally, the lack of comparative arm precludes our ability to determine whether weekly paclitaxel is superior, or at least as equivalent, to other agents including doxorubicin.

## Conclusion

In present retrospective study, we reviewed the efficacy and tolerability of first-line chemotherapy with weekly paclitaxel in unselected Korean metastatic angiosarcoma patients. We found weekly paclitaxel chemotherapy is effective in controlling metastatic angiosarcoma. It is encouraging that the response rate and PFS for Korean patients were similar to those demonstrated in prior Western reports.

## Abbreviations

STS: soft tissue sarcoma
RECIST: response criteria in solid tumors
ECOG: Eastern Cooperative Oncology Group
FNCLCC: Fédération Nationale des Centres de Lutte Contre le Cancer
